# Neurochemical and Neuropharmacological Aspects of Circadian Disruptions: An Introduction to Asynchronization

**DOI:** 10.2174/157015911795596522

**Published:** 2011-06

**Authors:** Jun Kohyama

**Affiliations:** Tokyo Bay Urayasu/Ichikawa Medical Center, 3-4-32 Toudaizima, Urayasu 279-0001, Japan

**Keywords:** Melatonin, serotonin, circadian rhythm, social jet lag, singularity, sleep hygiene, desynchronization.

## Abstract

Circadian disruptions are common in modern society, and there is an urgent need for effective treatment strategies. According to standard diagnostic criteria, most adolescents showing both insomnia and daytime sleepiness are diagnosed as having behavioral-induced sleep efficiency syndrome resulting from insomnia due to inadequate sleep hygiene. However, a simple intervention of adequate sleep hygiene often fails to treat them. As a solution to this clinical problem, the present review first overviews the basic neurochemical and neuropharmachological aspects of sleep and circadian rhythm regulation, then explains several circadian disruptions from similar viewpoints, and finally introduces the clinical notion of asynchronization. Asynchronization is designated to explain the pathophysiology/pathogenesis of exhibition of both insomnia and hypersomnia in adolescents, which comprises disturbances in various aspects of biological rhythms. The major triggers for asynchronization are considered to be a combination of light exposure during the night, which disturbs the biological clock and decreases melatonin secretion, as well as a lack of light exposure in the morning, which prohibits normal synchronization of the biological clock to the 24-hour cycle of the earth and decreases the activity of serotonin. In the chronic phase of asynchronization, involvement of both wake- and sleep-promoting systems is suggested. Both conventional and alternative therapeutic approaches for potential treatment of asynchronization are suggested.

## 1. INTRODUCTION

People whose circadian rhythms are out of phase with their daily schedule are said to have ‘social jet lag’ [1, 2]. Circadian rhythm problems are common in modern society, and there is an urgent need for effective treatment strategies. In Japan, approximately one quarter of junior high school students suffer from insomnia, while more than half of the students complain of daytime sleepiness [3]. Among adolescents in China, 16.1% had insomnia and 17.9% had daytime sleepiness [4]. In the United States, 22.8 % of adolescents reported insomnia and 34.7 % had daytime fatigue or sleepiness [5]. Among adolescents in Spain, the prevalence of insomnia was found to be 9.9% and that of daytime sleepiness, 53.0% [6]. These subjects had delayed bedtimes with complaint of insomnia. Delayed bedtimes result in sleep loss [7], producing daytime sleepiness. Daytime sleepiness could cause nocturnal insomnia. The subjects are living with disturbed habits such as delayed wake-up times, delayed bedtimes, and an irregular lifestyle. Speculatively, these subjects may be suffering from behavioral-induced sleep efficiency syndrome, presumably resulting from insomnia due to inadequate sleep hygiene. In fact, frequent complaints of these subjects introduced in my former review (Table **1**) [3] were compatible with associated features of behavioral-induced sleep-deficient syndrome [8], most likely due to inadequate sleep hygiene with low-level physical activity (sedentary behavior), excessive media exposure, and excessive caffeine use [9]. If this were the case, these symptoms should be ameliorated following adequate sleep hygiene (Table **2**). However, such therapeutic approaches often fail. Unknown factors or neuronal mechanisms may be preventing recovery. Asynchronization was proposed to solve this clinical problem [3], taking a basic concept of singularity (suppression of circadian rhythm) [10] into consideration. The present review first provides an overview of the basic neurochemical and neuropharmachological aspects of sleep and circadian rhythm regulation, then explains several circadian disruptions from similar viewpoints, and finally introduces the clinical notion of asynchronization.

## 2. BASIC ASPECTS OF SLEEP AND CIRCADIAN RHYTHM REGULATION

### 2.1. Basic Aspects of Sleep

Orexin, neuropeptide S (NPS), and histamine are known as representative neurotransmitters that participate in promoting arousal. Orexin-A levels in lumbar cerebrospinal fluid have been reported to be slightly higher at 5 AM than at 5 PM [11], whereas no report on diurnal fluctuation of NPS is available. Interestingly, NPS produces anxiolytic-like effects as well [12, 13]. Diurnal variation in cerebrospinal fluid levels of tele-methylhistamine (t-MH), the main histamine metabolite, was detected in children; the mean t-MH concentration was significantly higher during the daytime than during nighttime [14]. Compatible with this observation, mice histamine neurons in the tuberomammillary nuclei of the posterior hypothalamus were found to be active only during wakefulness [15]. Interestingly, the neurons’ activity varies at the different waking states, and is lowest during quiet waking, moderate during active waking, and highest during attentive waking. Their activity is likely to be related to a high level of vigilance. Histamine-1-receptor (H1R) agonists are known to be implicated in inducing arousal and wakefulness in humans, and in H1R knockout mice, the motivational effects of novelty are reported to be diminished [16]. 

The histaminergic system recently has been found to be affected by adenosine. Endogenous adenosine in the tuberomammillary nucleus, where histaminergic neurons exist, suppresses the histaminergic system *via *adenosine A1 receptors to promote non-rapid eye movement sleep [17]. Adenosine was also reported to mediate the sleep-promoting effect of prostaglandin D(2) through adenosine A2A receptors [18]. Caffeine is known to induce wakefulness by binding to adenosine A2A receptors as an antagonist [18], whereas the mechanism of action of modafinil, the antinarcoleptic, is still unknown [19]. In addition to adenosine [20], many sleep-promoting systems or substances have been reported. The ventrolateral preoptic area [21] and the median preoptic nucleus [22] are widely accepted areas to induce and/or maintain sleep. The discovery of the delta-sleep-inducing peptide [23] facilitated the progress of this research area. The studies on the sleep-modulating effects of cytokines [24] followed the discovery of the sleep-promoting effects of muramyl peptides [25]. Prostaglandin D(2) was most likely the endogenous sleep substance described by Ishimori [26] and Piéron [27] about 100 years ago [28]. In addition, uridine [29], oxidative glutathione [30], organic bromide compound [31], and fatty acid amide hydrolase [32] are reported to have sleep-promoting effects.

On the molecular level, several alterations associated with sleep have been reported. The number of synaptic terminals was reduced during sleep, but this decline was prevented by sleep deprivation [33] In the *Drosophila* brain, protein levels of key components of central synapses were found to be higher after waking and lower after sleep [34]. In humans, with regard to behavior, sleep loss has been demonstrated to exert a negative effect on daytime functions [35-37], general well being [38], metabolic and endocrine function [39, 40], body weight [41], viral infections [42], and psychomotor vigilance, including mood [43]. However, the required sleep duration of an individual person is difficult to determine because the need for sleep is variable and depends on many factors [44]. In addition, regardless of sleep duration, problematic behaviors are known to be associated with the aforementioned disturbances in daily habits [45-57].

The duration of sleep is longer in the winter than in the summer [58], and the adolescents in East Germany have earlier waking times and bedtimes than those in West Germany [59]. Human sleep behavior is supposed to be regulated by sunlight, although unconsciously.

### 2.2. Basic Aspects of Circadian Rhythm

In mammals, intrinsically photosensitive retinal ganglion cells (ipRGCs) exhibit light responses independent of rod and cone signaling [60, 61]. These cells express the photopigment melanopsin, project directly to the suprachiasmatic nucleus (SCN) of the hypothalamus (the site of the circadian clock), and thereby contribute to non-image-forming responses to light. Light stimuli activate N-methyl-d-Aspartate (NMDA)/non-NMDA receptors of the SCN [62]. Signals from the SCN regulate various circadian rhythms including feeding, locomotion, sleep-wake alternation, corticosterone secretion [63], and the autonomic nervous system [64]. Typically, the endogenous period of the circadian clock in humans is longer than 24 hours, although the racial differences in the human endogenous circadian period have been reported [65]. It is through morning exposure to sunlight that people become accustomed to the 24-hour cycle [66]. Conversely, light exposure at night delays the circadian clock phase [66] or disrupts its function [67, 68]. Thus, nocturnal light exposure has unfavorable effects on the biological clock. Non-photic cues such as physical activity [69], social factors [70], and eating times [71] also serve to synchronize the circadian system to a 24-hour day. Of these, the mechanism of food-anticipatory rhythms has recently been clarified [71]: The dorsomedial hypothalamic nucleus was determined to be a putative food-entrainable circadian pacemaker in mice, and oscillation of this pacemaker was found to persist for at least two days, even when mice received no food during the expected feeding period following establishment of food-entrained behavioral rhythms.

In the absence of time cues, daily rhythms become altered and develop their own rhythm. After living under such unusual conditions for a considerable period of time, reciprocal phase interactions within circadian rhythms such as sleep-wakefulness and temperature are disturbed [72]. In general, most people wake in the morning when their body temperature begins to rise from its lowest level and, conversely, fall asleep at night when their body temperature begins to decline from its highest level. However, once this reciprocal interaction is impaired, the phase relationship between body temperature and sleep-wakefulness circadian rhythms is disrupted [72]. This disruption is termed ‘circadian desynchronization’, and produces various physical and mood disturbances such as disturbed nighttime sleep, impaired daytime alertness and performance, disorientation, gastrointestinal problems, loss of appetite, inappropriate timing of defecation, and excessive need to urinate during the night [73-75]. Similar complaints and mood alterations have been observed in patients with jet lag [76] or seasonal affective disorder [77], and in astronauts [78]. 

‘Larks’ and ‘owls’ are respective terminologies for people who wake up early in the morning and are ready for sleep early in the evening, and those who wake up late in the morning and feel less sleepiness even late in the evening. A self-assessment questionnaire was used to determine if an individual was a morning- or an evening-type person (chronotypes: larks or owls) [79]. Endogenous phasing of the circadian biological clock in morning-type individuals varies from that of evening-type individuals [80]; the latter experience a temperature rise later in the morning and later waking times [81]. Moreover, individuals who are alert in the morning experience an earlier circadian rhythm temperature peak than individuals who are alert in the evening [82]. Furthermore, it was suggested that evening-type individuals suffer from circadian desynchronization [74, 75]. 

Interestingly, it was previously demonstrated that kidney cells followed a 24.5-hour clock, whereas corneal cells ran at approximately 21.5 hours [83], suggesting that peripheral clocks may work in different circadian cycles. The SCN might serve as a reference point for the peripheral clocks, and the SCN and the peripheral clocks can be considered to work under unstable synchrony. The loss of synchrony between body tissues could underlie some of the health problems seen in circadian-rhythm disorders and shift workers [1]. In a recent study examining the molecular circadian rhythms of 11 larks and 17 owls, as assessed from the expression of brain and muscle Arnt-like protein-1 (Bmal1) in their skin cells, cells from larks showed shorter circadian period length than those from owls [84]. Bmal1 is a transcription factor known to regulate circadian rhythm [85]. Approximately half of the larks and owls actually had ‘normal’ circadian period length, whereas the owls had skin clocks that were more difficult to reset than those of people with more typical schedules, and the larks had clocks that were easier to reset [84]. These studies suggest that individual differences in chronotype can result from both innate differences in circadian period length and in how easily a person’s rhythms can be synchronized to the night-day cycle.

### 2.3. Melatonin

Melatonin regulates the circadian phase [86], acts as a hypnotic, and is an effective free-radical scavenger and antioxidant, which directly induces gonadotropin-inhibitory hormone expression [87]. In addition, melatonin is considered to be involved in the inhibition of cancer development and growth [88]. Interestingly, bright light during the nighttime decreases melatonin secretion [89]. Melatonin immune staining was demonstrated in the bacterium *Rhodospirillum rubrum*, one of the oldest species of living organisms (potentially 2-3.5 billion years) [90]. Bacterial melatonin might provide on-site protection of bacterial DNA against free-radical attack. Melatonin is also known to exert antioxidant effects in the brain [91], and sleep is hypothesized to function as an antioxidant or scavenging process in the brain [92]. 

Melatonin promotes and synchronizes sleep by acting on SCN-expressing melatonin MT1 and MT2 receptors, respectively. Synthesized melatonin receptor agonists exhibiting an increased duration of action are expected to provide significant clinical value for treatment of insomnia patients [93]. The onset of melatonin secretion begins 14-16 hours after waking, usually around dusk [94]. Exposure to bright, mid-day light has been shown to increase melatonin secretion during the night, without a circadian phase shift [95]. In a preliminary study of 3-year-old children, early sleepers tended to exhibit higher levels of urinary 6-sulfatoxymelatonin (6SM) (6SM/creatinine ratio), the primary melatonin metabolite, compared with late sleepers [96]. Decreased melatonin levels in aged zebrafish have been shown to correlate with altered circadian rhythms [97]. An inversion in melatonin circadian rhythm secretion was also observed in alcoholics, not only during alcohol intake but also during short- and long-term withdrawal [98]; the authors concluded that circadian disorganization of melatonin secretion may be responsible for desynchronization in some alcoholic patients. Because melatonin regulates the circadian phase [86], altered melatonin secretion could disturb circadian oscillation, producing various biological alterations. Nevertheless, in the rat, altered melatonin rhythm was reported to have no effect on circadian rhythms of locomotor activity and body temperature [99]. 

Of the synthesized ligands of melatonin receptors [100], agomelatine for the treatment of depression [101], ramelteon for the treatment of primary chronic insomnia characterized by difficulty with sleep onset [102], and tasimelteon for transient insomnia in circadian rhythm sleep disorders [103] are the three clinically important agents. Agomelatine especially represents a new concept for the treatment of depression [104]. The antidepressant activity of agomelatine does not depend solely on its agonistic action at melatonergic receptors, but also on its antagonistic activity at type 2C serotonin (5-HT2C) receptors. Agomelatine also exhibits anxiolytic properties that bear a striking resemblance to those of selective 5-HT2C receptor antagonists. In addition, agomelatine rapidly regulates the sleep-wakefulness cycle without causing sedation and improves daytime condition. This compound may open new aspects on the pathophysiology of depression [104]. 

### 2.4. Serotonin

Serotonergic activity is highest during wakefulness, decreases during non-rapid eye movement sleep, and almost ceases during rapid eye movement sleep [105]. Involvement of serotonin in circadian rhythm formation has recently been shown. Kennaway *et al*. showed that activation of 5-HT2C, similar to the effect of a light pulse, produces long-lasting phase shifts in melatonin rhythm and that this effect is blocked by 5-HT2C antagonist [106]. Serotonin is also summarized to advance the phase of master pacemaker oscillation when applied during the subjective day and to inhibit light-induced phase shifts during the subjective night [107].

The serotonergic system is activated not only through exposure to morning light [108] but also through rhythmic movements, such as gait, chewing, and respiration [109]. Exercise-derived benefits for brain function have been demonstrated at the molecular level [110], and physical activity has been reported to decrease the risk of Alzheimer’s disease [111, 112]. Physical activity is one of the key factors promoting brain function in animals and humans. Serotonin is considered one of the important agents that mediate these exercise-induced effects [113].

The concept of low serotonin syndrome, which comprises aggressiveness, impulsivity, and suicidal behavior, has been proposed previously [114]. In male adult vervet monkeys, decreased serotonergic activity was reported to be a disadvantage for attaining high social dominance status, whereas enhanced activity was advantageous [115]. Disturbance in the lateral orbito-prefrontal circuit induces aggressive behavior and loss of sociability [116], and the serotonergic system has been shown to activate this circuit [117]. Serotonin levels have also been shown to enhance learning ability [111]. Results from positron-emission tomography scanning and alpha-[(11)C]methyl-L-tryptophan trapping suggest that low serotonin synthesis in the prefrontal cortex lowers the threshold for suicidal behavior [118]. In humans, Schweighofer *et al.* found that an impairment in the serotonergic system is linked to action choices that are less advantageous in the long run [119]. Since serotonergic activity is profoundly affected by the sleep-wakefulness cycle [105], these studies raised the possibility that irregular sleep-wakefulness rhythm disturbs emotional control, sociability, and also perspective because of decreased serotonergic activation.

## 3. REPORTED CONDITIONS SHOWING CIRCADIAN DISRUPTION

For the trans-meridian traveler, both physical cues such as daylight and darkness, and social cues such as mealtimes and noise, encourage realignment of the circadian system. By contrast, for the shift worker, physical cues are resolutely opposed to nocturnal alignment, as are most social cues stemming from a day-oriented society. Therefore, circadian realignment of shift workers takes longer than realignment from jet lag [120]. A report from the World Health Organization's International Agency for Research on Cancer concluded that “shift-work that involves circadian disruption is probably carcinogenic to humans” [121]. Reduced activity of the melatonergic system is suggested to be a causative factor for this association [122]. In addition, a forced, extraordinary schedule can induce desynchronization [70]. The modern ‘24/7’ lifestyle with wide spread use of artificial bright light could also produce shift work disorder or social jet lag. Furthermore, a quarter of the world’s population is subjected to a one-hour time change twice a year (daylight saving time; DST) [58]. DST is now known to disturb the normal seasonality seen in sleep timing as assessed by mid-sleep times [58]. In addition, at the beginning of DST (i.e., spring), the rates of traffic accidents [123] and myocardial infarctions [124] increase. DST can be described as a system that produces social jet lag in a huge number of people.

Altered circadian rhythms have been described in childhood chronic fatigue syndrome (CFS), in which the patients suffer from an atypical, but continuous, jet lag condition [125]. One third of children with CFS exhibited abnormal cardiovascular regulation during postural changes (orthostatic dysregulation), as characterized by instantaneous orthostatic hypotension, or postural or neural-mediated syncope [126]. Compared with the control population, adult patients with CFS had significantly lower systolic blood pressure and mean arterial blood pressure as well as exaggerated diurnal variation [127]. People with CFS were also reported to demonstrate lower salivary cortisol concentrations in the morning and higher salivary cortisol concentrations in the evening compared with people who did not have CFS and with non-fatigued controls, indicating a flattening of the diurnal cortisol profile [128]. However, studies in adults suggest that circadian rhythm is disturbed in some patients with CFS. A British cohort study revealed that sedentary behavior during childhood increased the risk of CFS/myalgic encephalomyelitis, for which depression is a major symptom [129]. Selective serotonin reuptake inhibitors, which are widely used for patients with depression, have been reported to be effective in treating patients with CFS [130], in which decreased serotonergic activity is considered to be involved. Melatonin has also been shown to be effective for CFS patients with delayed circadian rhythm [131]. 

The characteristic clinical burnout symptoms include excessive and persistent fatigue, emotional distress, and cognitive dysfunction [132]. These symptoms are common among disorders such as depression and CFS [133]. Compared with controls, burnout cases had similar morning salivary cortisol levels and a similar awakening response, but lower evening cortisol, suggesting a flattening of the diurnal cortisol profile [134]. Subjects exhibiting burnout are reported to exhibit a higher frequency of arousal during sleep [133]. A study of university hospital nurses revealed that daylight exposure for at least three hours per day resulted in reduced stress and greater job satisfaction, both of which were favorable factors for reducing burnout [135]. Because bright, midday light increases melatonin secretion during the night in elderly individuals [95], the melatonergic system might be involved in the pathogenesis of burnout. A possible involvement of the serotonergic system in burnout has also been indicated [136].

Fibromyalgia is characterized by wide spread pain and muscle tenderness, as determined by palpation, lasting at least three months [8]. In patients with fibromyalgia, a significant delay in the rate of decline of cortisol levels from acrophase to nadir was reported [137]. A serotonin and norepinephrine reuptake inhibitor was used to successfully treat patients with fibromyalgia [138], whereas melatonin was also able to reduce the pain associated with this syndrome [139]. Physical activity is also known to show favorable effects on fibromyalgia [140], suggesting involvement of the serotonergic system. 

Decreased circadian rhythm amplitude has been reported in the more common conditions of mood disorders, including major depressive disorder [141-143], for which serotonin reuptake inhibitors are used as antidepressant treatment. Furthermore, a melatonin receptor agonist was shown to exhibit a favorable effect on depression [104].

From a therapeutic point of view, decreased serotonergic and melatonergic activity is suggested in CFS, burnout, fibromyalgia, and depression, although each of these conditions possesses its own specific origin, major symptoms, course, and age of onset.

## 4. REQUIREMENT FOR A NOVEL CLINICAL ENTITY

Although delayed wake-up times and bedtimes could be symptoms of a delayed sleep phase form of a circadian rhythm sleep disorder, it should be noted that the biological- and lifestyle-related sleep phase delays that are especially common during adolescence are often misdiagnosed as this disorder [144]. According to the diagnostic criteria, patients with circadian sleep disorders of delayed sleep phase type or irregular sleep-wakefulness type should exhibit normal sleep duration for their age [8]. But the sleep duration of many of the young people in Japan who exhibit intractable circadian rhythm disruptions is essentially decreased (or varies day by day), and results in complaints of both insomnia and hypersomnia. Similarly, sleep insufficiency on weekdays was emphasized in Chinese adolescents [4]. In Taiwan, increased academic workloads are thought to lead to a delayed bedtime and short nocturnal sleep duration among adolescents [145]. These adolescents were not diagnosed with circadian rhythm sleep disorder of delayed sleep phase type, but the need for education on adequate sleep hygiene was emphasized [145]. Many adolescents are suffering from disturbed circadian rhythms with decreased sleep duration. These young people are hard to diagnose as having various types of circadian sleep disorders on the basis of diagnostic criteria [8]. Another issue of note is that the risks for behavioral and metabolic aspects of these sleep disorders have been increased, despite wide recognition of the need for education on sleep hygiene [146]. It is possible that certain unknown factors other than simple sleep loss and inadequate sleep hygiene are involved in many of these problematic adolescents. 

In 1970, Winfree reported that a specific, dim, blue-light pulse stimulus, with a unique stimulus time and duration, resulted in unusual broadening of the daily eclosion peaks of the fruit fly, *Drosophila pseudoobscura*, even to the extreme of obscuring circadian rhythm [10]. This phenomenon, suppression of circadian rhythms, was called ‘circadian singularity behavior’ [147], and has since been described in a range of organisms including algae, plants, and mammals [147, 148]. In humans, circadian rhythms of rectal temperature and plasma cortisol were reported to be abolished by a single, long-duration bright-light pulse administered during one or two successive circadian cycles [148]. A temperature increase and light pulses were shown to trigger circadian singularity behavior in *Neurospora *circadian clock gene frequency [147], whereas a critical light pulse (three-hour light pulses delivered at near subjective midnight) was shown to drive cellular clocks to singularity behavior in mammals [68]. Interestingly, this phenomenon is transient [147], although removal of the stimulus is required. These findings taken together with the basic entity of singularity designated a clinical concept termed ‘asynchronization’. The aim of this designation essentially was to explain the aforementioned disturbed conditions of adolescents, assuming that the function of the circadian clock of these young people is markedly impaired and might be lost. 

## 5. ASYNCHRONIZATION

Asynchronization is the result of disturbed aspects (e.g., cycle, amplitude, phase, and interrelationship) of biological rhythms that normally exhibit circadian oscillation, which presumably involve decreased serotonergic and/or melatonergic activity. The major trigger of asynchronization is considered to be a combination of light exposure during night-time, which reduces melatonin secretion, and a lack of morning light exposure, which decreases serotonergic activity [3]. Both triggers are known to disturb the biological clock in the SCN [66]. Asynchronization symptoms (Table **3**) include disturbances of the autonomic nervous system (sleepiness, insomnia, disturbed hormonal secretion, gastrointestinal problems, and sympathetic nervous system predominance), as well as disturbances of higher brain functions (disorientation, loss of sociability, loss of will or motivation, and impaired alertness and performance). Neurological (attention deficits, aggression, impulsiveness, and hyperactivity), psychiatric (depressive disorders, personality disorders, and anxiety disorders), and somatic (tiredness, fatigue, neck and/or back stiffness, and headache) disturbances are also putative symptoms of asynchronization.

Among disturbances of the autonomic nervous system, asynchronization, over-activation of the sympathetic nervous system [149, 150] and disturbed hormonal secretion due to activation of the hypothalamic-pituitary-adrenal axis [151, 152] have been reported in the hyperarousal model of primary insomnia. Asynchronization and primary insomnia might share a common pathophysiological background. It is likely that activation of the hypothalamic-pituitary-adrenal axis is involved in asynchronization. Irregular bowel habits are one of the representative gastrointestinal symptoms of asynchronization. Among healthy adults, an increase of colonic pressure resulting in emptying of the colon is observed in the morning and during the two hours after meals [153], with those with regular bowel habits more likely to defecate in the morning [154]. Seventy-seven percent of the adult non-patient population in Beijing, for instance, was reported to defecate in the morning [155]. Bowel habits are under circadian control, and in normal circadian oscillation, defecation is expected to be seen regularly in the morning. Bowel habits are likely to be easily affected with circadian disruptions. Indeed, irritable bowel syndrome (IBS) and CFS are reported to share predisposing risks, suggesting a common predisposing pathophysiology [156]. Similarly, frequent overlapping of IBS with conditions showing circadian disruption (i.e., CFS and fibromyalgia) has been known and the question of a common underlying pathophysiology has been raised [157]. 

**Fig. (1).**
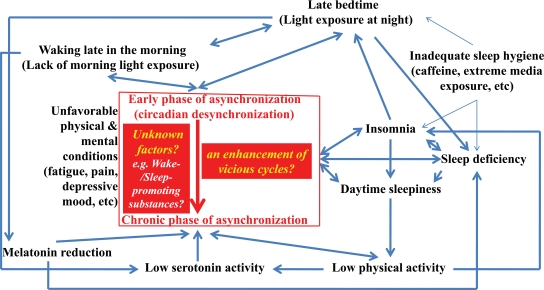
**Schematic drawing of the development of asynchronization.** The broad blue lines constitute vicious circles, and enhancement of these circles is involved in entering into the chronic phase of asynchronization. Certain unknown factors (broad red line) might act as promoters for progression from the early phase into the chronic phase of asynchronization.

The early phase of asynchronization or the state of circadian desynchronization is easily induced by the described disturbances in daily habits, producing unsatisfactory physical, mental, and/or emotional conditions, presumably leading to decreased physical activity. This condition is considered to be functional and can be resolved relatively easily by establishing a regular sleep-wakefulness cycle. However, without adequate intervention, the disturbances can gradually worsen, resulting in a further decrease in physical activity and also in serotonergic and/or melatonergic activity, which can be difficult to resolve (Fig. **1**). Patients in the chronic phase of asynchronization present daily sleep durations of more than 10 hours three times or more in a week, despite elevation of sympathetic nervous system activity. Regulation of both wake- and sleep-promoting systems might be disturbed in the chronic phase of asynchronization. 

Development of the clinical phases of asynchronization might be depend on individual differences not only in the ease of rhythm synchronization to the night-day cycle [84] but also in the endogeneous period of the circadian clock [65]. In addition, certain unknown factors (broad red line) might act as promoters of development of the chronic phase of asynchronization (Fig. **1**). So far, neither preschoolers nor adults who have developed the chronic phase of asynchronization have been reported. Thus, age of adolescence might be one of the promoting factors.

Pain is a common symptom in depression, fatigue, and circadian disruption with or without insomnia/hypersomnia [158] as well as in asynchronization, although the underlying cause of the pain remains to be determined. Pain is one of the diagnostic criteria for childhood CFS [159]; furthermore, a relationship between burnout and musculoskeletal pain [160] and a 30-60% co-occurrence rate of depression and pain [161] have been reported. Regarding these associations, Maletic and Raison [158] emphasized the role of dysregulation of stress/inflammatory pathways. In that study, this functional change was suggested to promote alterations in brain circuitry that modulates mood, pain, and the stress response, resulting in disruptions in neurotrophic support and disturbances of glia-neuronal communication, especially when patients were inadequately treated. This presumable situation could also be involved in the chronic phase of asynchronization. 

## 6. POTENTIAL THERAPEUTIC APPROACHES FOR ASYNCHRONIZATION

### 6.1. Basic Principles

For synchronization of the biological clock to a 24-hour cycle, exposure to morning light and avoidance of nocturnal light are essential. Therefore, failure to practice these two behaviors will be risk factors to develop asynchronization. Moreover, light-induced adrenal gene expression and corticosterone release have been demonstrated [162]. Under normal conditions, steroid secretion is greatest in the morning. As described earlier in this article, physical activity [69], social factors [70], and eating time [71] are known to affect the circadian clock. Adequate physical activity (exercise), participation in social activities, and regular mealtimes are likely to prevent asynchronization. A daytime nap is known to result in favorable performance [163]. However, evening-type adolescents were reported to nap more frequently during school days than other chronotypes [52], although improved school performance after an afternoon 15-minute nap was reported in a Japanese high school [164]. Further studies are required to determine whether napping affects asynchronization. Overall, for prevention of asynchronization, social promotion of favorable sleep hygiene including health education beginning in early elementary school age is important [165, 166]. 

Decrease of serotonergic and/or melatonergic activity is considered essential pathophysiology of the chronic phase of asynchronization (Fig. **1**). Interestingly, selective serotonin reuptake inhibitor (fluvoxamine) and selective noradrenalin-serotonin reuptake inhibitor (desipramine) increased evening plasma melatonin concentrations and prolonged the duration of elevated melatonin secretion, respectively [167]. Desipramine, but not fluvoxamine, increased urinary 6SM excretion. It is suggested that the elevated plasma melatonin observed following fluvoxamine is caused by inhibition of CYP1A2-mediated melatonin metabolism [167]. The unknown common pathway in which both serotonergic systems and melatonergic systems are involved might be implicated in the chronic phase of asynchronization. 

### 6.2. Conventional and Alternative Approaches

Melatonergic [100, 104, 131, 139] and serotonergic [104, 130, 138, 143] agents as well as hypnotics, antidepressants, vitamin B12, physical activity [140], chronotherapy, and bright light in the morning or during the daytime [168] and darkness during nighttime [169] are conventional approaches used to treat asynchronization. Low dose sulpiride, known to exert antidepressant activity through the blockade of D2/D3 receptors [170], might be another promising choice for the treatment. 

Potential alternative approaches include traditional Chinese medicine, direct contact to the body (e.g.; hug), autonomic nervous system control, pulse light therapy, and rhythmic movements such as qigong, tanden breathing, and locomotion [3]. 

In addition, favorable effects of acupuncture for depression [171, 172], fibromyalgia [173], insomnia [174], and CFS [175], and of shiatsu (acupressure) for sleep disturbance [176] and CFS [177] were reported. These may also be promising therapeutic tools for the treatment of asynchronization. However, their diagnostic standards and methodology as treatment strategies remain to be determined.

## 7. CONCLUSION

This review introduced the clinical notion of asynchronization, focusing on the association between nocturnal lifestyle and biological clock disorders, as well as on the melatonergic and serotonergic systems. There is also potential for the involvement of dopamine [170, 178], neuropeptide Y [107], and opioid peptides [125] in the pathogenesis of asynchronization. 

Famous traditional wisdoms [179, 180] also recommend morning-type behaviors to have healthy lives. In 1879, Thomas Alva Edison developed a long-lasting, practical electric light bulb. At that time, people were wondering whether human beings could be awake 24 hours a day. Unfortunately, the current social trend of 24/7 activity may produce unfavorable effects on the SCN, given that the negative effects of exposure to nocturnal light on human beings are well established [1, 66-68, 89]. Future studies are required on the detailed properties of the biological clock, with the aim of identifying treatments for patients suffering from circadian disruptions. 

## Figures and Tables

**Table 1.** Frequent Complaints of Youngsters in Japan*

Elementary School Students		Junior High School Students	Boys/Girls
Persistent need to yawn	62%	Desire to sleep	73.8%/80.8%
Desire to sleep	58%	Persistent need to yawn	43.6%/69.1%
Desire to lie down	47%	Desire to lie down	43.2%/47.2%
Eyestrain	33%	Eyestrain	40.7%/44.7%
Difficulty in sitting straight	29%	Difficulty with memorization	35.2%/33.6%
Difficulty with memorization	28%	Neck stiffness	29.3%/35.1%
Irritation	27%	Lumbago	26.5%/23.2%
Neck stiffness	26%	Low activity	21.3%/28.0%
Low activity	25%	Hypersensitivity	20.0%/27.0%
Difficulty in concentrating	25%	Difficulty in concentrating	21.0%/23.8%
Hypersensitivity	24%	Irritation	20.5%/24.2%
Thirst	21%		
Making many mistakes	20%		

* Only items comprising more than 20% of complaints are listed.

**Table 2.** Requirements for Adequate Sleep Hygiene

Issues	Examples
Morning light exposure	
Physical activities	
Avoidance of nocturnal light exposure	
Regular meals	
Avoidance of inappropriate substances	Caffeine, nicotine
Suitable environment	Illumination, temperature, humidity, noise

**Table 3.** Features of Asynchronization

	Concepts	Details
Essence	Disturbance of various aspects of biological rhythms (e.g., cycle, amplitude, phase, and interrelationship) that indicate circadian oscillation	
Presumable causes	Light exposure during the night	
	Lack of light exposure in the morning	
	Decreased physical activity	
	Disturbance of biological clock and/or serotonergic/ melatonergic systems	
Symptoms	Disturbances related to the autonomic nervous system	Sleepiness, insomnia, disturbance of hormonal secretion, gastrointestinal problems, sympathetic nervous system predominance
	Somatic disturbances	Tiredness, fatigue, neck and/or back stiffness, headache, persistent yawn, desire for sleep, wish to lie down, inactivity, lumbago
	Disturbances related to higher brain function	Disorientation, loss of sociality, loss of will or motivation, impaired alertness and performance, difficulty with memory or concentration
	Neurological disturbances	Attention deficit, aggression, impulsiveness, hyperactivity, irritation, hypersensitive
	Psychiatric disturbances	Symptoms observed in depressive disorders, personality disorders, and anxiety disorders
Therapeutic approaches	Morning light exposure	
	Avoidance of nocturnal light exposure	
	Conventional approaches	Light therapy, medications (hypnotics, antidepressants, melatonin, etc.), physical activation, chronotherapy
	Alternative approaches	Traditional Chinese medicine, qigong, tanden breathing, acupuncture
Prognosis	Early phase (functional?)	Can be easy to resolve
	Chronic phase	Difficult to resolve
